# Chlamydia related bacteria (*Chlamydiales*) in early pregnancy: community-based cohort study

**DOI:** 10.1016/j.cmi.2016.10.011

**Published:** 2017-02

**Authors:** F. Reid, P. Oakeshott, S.R. Kerry, P.E. Hay, J.S. Jensen

**Affiliations:** 1) Department of Primary Care and Public Health Sciences, King's College London, London, UK; 2) Population Health Research Institute, St George's, University of London, London, UK; 3) Courtyard Genitourinary Medicine Clinic, St George's NHS Trust, London, UK; 4) Statens Serum Institut, Copenhagen, Denmark

**Keywords:** *Chlamydiales*, Cohort study, Miscarriage, Pregnancy, Preterm birth, Prevalence

## Abstract

**Objectives:**

Serological case–control studies suggest that certain chlamydia-related bacteria (*Chlamydiales*) which cause cows to abort may do the same in humans. *Chlamydiales* include *Waddlia chondrophila*, *Chlamydia abortus* and *Chlamydia trachomatis.* Data on prevalence of *Chlamydiales* in pregnancy are sparse. Using stored urine samples from a carefully characterised cohort of 847 newly pregnant women recruited from 37 general practices in London, UK, we aimed to investigate the prevalence and types of *Chlamydiales* infections. We also explored possible associations with miscarriage or spontaneous preterm birth.

**Methods:**

Samples were tested using *W. chondrophila* and pan-*Chlamydiales* specific real-time PCRs targeting the 16S rRNA gene. Samples positive on either PCR were subjected to DNA sequencing and *C. trachomatis* PCR.

**Results:**

The overall prevalence of *Chlamydiales* was 4.3% (36/847, 95% CI 3.0% to 5.8%). The prevalence of *W. chondrophila* was 0.6% (*n* = 5), *C. trachomatis* 1.7% (*n* = 14), and other *Chlamydiales* species 2.0% (*n* = 17). Infection with *C. trachomatis* was more common in women aged <25, of black ethnicity or with bacterial vaginosis, but this did not apply to *W. chondrophila* or other *Chlamydiales.*

Follow up was 99.9% at 16 weeks gestation and 90% at term. No infection was significantly associated with miscarriage at ≤12 weeks (prevalence 10%, 81/827) or preterm birth <37 weeks (prevalence 4%, 23/628). Of 25 samples sequenced, seven (28%) were positive for *Chlamydiales* bacterium sequences associated with respiratory tract infections in children.

**Conclusion:**

In the first study to use the pan-*Chlamydiales* assay on female urine samples, 4% of pregnant women tested positive for *Chlamydiales*, including species known to be pathogenic in mothers and neonates.

## Introduction

Each year in England and Wales around 100 000 women suffer a miscarriage and 50 000 have a preterm birth (<37 weeks gestation) at an annual estimated cost of over £300 million. Around 15% of miscarriages before 13 weeks gestation, 60% of later miscarriages and 40% of preterm births are associated with infection [1]. Serological case–control studies suggest that certain chlamydia-related bacteria (*Chlamydiales*) that cause cows to miscarry [2] may do the same in humans [3], [4]. The *Chlamydiales* order includes *Waddlia chondrophila*, *Chlamydia abortus* and *Chlamydia trachomatis.* The infections can be treated with azithromycin, which is safe in pregnancy [5]. However, their prevalence in pregnant women has never been assessed using a pan-*Chlamydiales* assay in urogenital samples.

We collected baseline first-void urine samples from a carefully characterized cohort of 1216 consecutive pregnant women recruited at <10 weeks gestation from 37 London urban general practices in 1998–2000 [6]. Integrity of bacterial DNA after prolonged storage at or below −30°C was confirmed in 2011 by repeat testing of the six *Mycoplasma genitalium* positive samples [7], including three with a low load of organisms (<5 copies/test). Previous studies from the cohort [8] and from pregnant women in the USA [9] showed the sensitivity of urine samples for *C. trachomatis* detection during pregnancy was comparable to that of self-taken vaginal swabs or endocervical samples.

Our main aim was to investigate the prevalence and types of *Chlamydiales* in stored urine samples from early pregnancy. We also explored whether infected women were more likely to miscarry or have a preterm birth than uninfected women. Finally, we used sequencing to analyse *Chlamydiales* not previously found in the urogenital tract of newly pregnant women.

## Materials and methods

The study was approved by Wandsworth Research Ethics Committee (reference 96.68.6) and participants gave informed consent. At recruitment the women completed questionnaires including demographic details and obstetric history, and provided first-void urine samples [6]. They were followed up by postal or telephone questionnaire (backed by medical record search for non-responders) asking about pregnancy outcome at 16 weeks and at term [10]. In 2014–15, stored urine samples were tested using validated *W. chondrophila* [11] and pan-*Chlamydiales* [12] specific real-time PCRs targeting the 16S rRNA gene. We performed the *W. chondrophila* PCR on all samples (rather than only those positive on the pan-*Chlamydiales* PCR) to optimize the *W. chondrophila* detection rate.

In brief, 5 μl of DNA lysate prepared by boiling pellets from spun urine in a Chelex (Bio-Rad, Hercules, CA, USA) slurry as previously described [13] was analysed in duplicate in a total reaction volume of 50 μl. The real-time PCRs included an internal control for inhibition [13] and positive controls comprised purified recombinant plasmids containing the 16S genes of *Parachlamydia acanthamoebae* [12] in the pan-*Chlamydiales* assay [12] and *W. chondrophila* [11] generously provided by Prof. Gilbert Greub, University of Lausanne, Lausanne, Switzerland. Samples positive in the pan-*Chlamydiales* PCR were sequenced using internal primers covering a 162 to 170 bp sequence depending on the species (primers excluded) [12] and tested for *C. trachomatis* by real-time PCR [14]. Sequences were searched against NCBI GenBank and the match with the highest score was noted for each sequence. If several sequences had the same score, the source of the sequences was checked and sequences reported from human samples were selected. Taxonomic assignment to the genus level was carried out using the Ribosomal Database Project Naive Bayesian rRNA Classifier Version 2.10.

### Sample size and statistical analysis

We were restricted by the size of the cohort, which was originally powered to investigate the association between bacterial vaginosis and miscarriage [6]. Prevalences are presented with 95% CI. Outcomes were compared between infected and uninfected women using two-sided Fisher's exact tests at a 5% significance level. We focused on early miscarriages at ≤12 weeks gestation because *W. chondrophila*-positive serology has been shown to be associated with early miscarriages [3] but not with late miscarriages after 12 weeks. Numbers did not permit adjustment for possible confounders. Statistical analyses were performed using Stata version 13, with exact confidence intervals calculated by Confidence Interval Analysis software version 1.2.

## Results

**Fig. 1:**
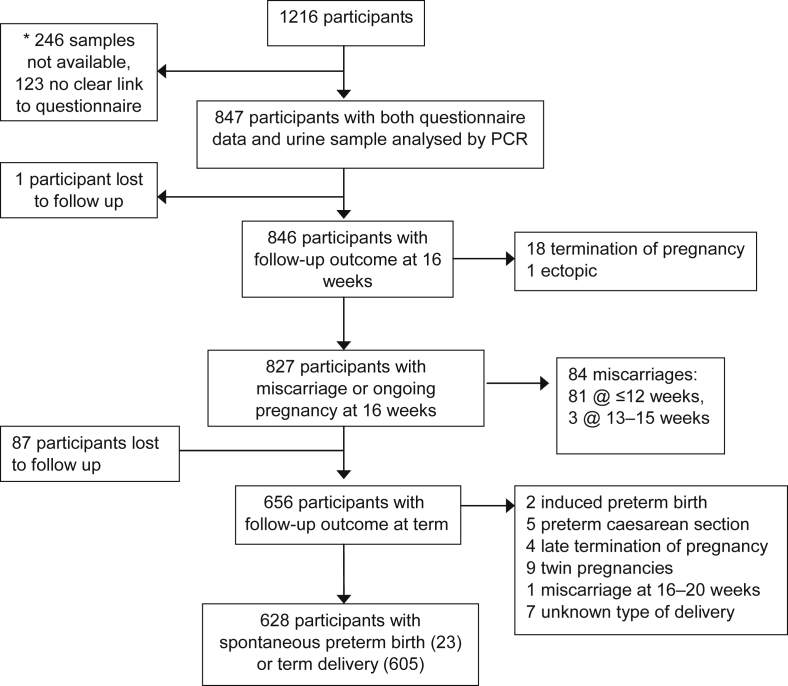
*Chlamydiales* flow chart. *Samples were collected in 1998–2000 and labelled by hand with ID number and date of birth. By 2013–14, after 15 years of storage, some labels were difficult to read, some containers were empty and some were missing. From 1216 participants we analysed 847 samples (70%) that had a clear ID number and date of birth that matched details on the questionnaire.

Urine samples from 847 (70%) women were available for analysis (Fig. 1). The mean age of the whole cohort of women was 31 years (range 16 to 46 years), 10% were of black ethnicity (Black Caribbean *n* = 48, Black African *n* = 30), 10% smoked during pregnancy, 40% were from social class 3–5 on the Standard Occupational Classification [6], [15], and 4% were teenagers (aged <20 years). Age, ethnicity and other characteristics were similar in included and excluded women (see Supplementary material, Table S1).

### Prevalence of *Chlamydiales*

The overall baseline prevalence of *Chlamydiales* including *W. chondrophila* and *C. trachomatis* was 4.3% (36/847, 95% CI 3.0%–5.8%). Prevalences of *W. chondrophila* and *C. trachomatis* were 0.6% (5/847, 95% CI 0.2%–1.4%) and 1.7% (14/847, 95% CI 0.9%–2.8%), respectively. No woman had both infections. Two of the five samples that were positive on the *W. chondrophila* PCR were negative on the pan-*Chlamydiales* PCR. These two samples were counted as *W. chondrophila* positives, because the specific *W. chondrophila* PCR assay was expected to have a higher sensitivity than the pan-*Chlamydiales* assay. The prevalence of *Chlamydiales* other than *W. chondrophila* and *C. trachomatis*, was 2.0% (17/847, 95% CI 1.2%–3.2%).

Infection with *C. trachomatis* was more common in women aged <25 years, of black ethnicity or with bacterial vaginosis (p <0.001) but this did not apply to *W. chondrophila* or other *Chlamydiales* (Table 1). The detailed characteristics of the five women with *W. chondrophila*-positive samples are given in Table 2.

### *Chlamydiales* and miscarriage or preterm birth

Information on outcome was available for 99.9% (846/847) of included women at 16 weeks gestation and for 90% (759/847) at term. After exclusions such as termination of pregnancy (Fig. 1), 827 women were analysed for miscarriage and 628 for preterm birth. No infection was significantly associated with miscarriage at ≤12 weeks gestation (*n* = 81, Table 3) nor with spontaneous preterm birth (*n* = 23, Table 4). One of three *W. chondrophila* positives, who were followed up to delivery, had a preterm birth compared with 4% (22/625) of uninfected women, but numbers were too small to confirm an association. Both of the women with an adverse pregnancy outcome who were positive on the *W. chondrophila* PCR were also positive on the pan-*Chlamydiales* PCR (see Supplementary material, Table S2).

### Admission to a special care baby unit (SCBU)

We also explored whether infection with *Chlamydiales* at <10 weeks gestation was associated with neonatal admission to SCBU. Rates of admission to SCBU were similar in babies from infected and uninfected women: 10% (2/20) versus 7.7% (39/508).

### Sequencing

Sequences were obtained from 25 (69%) of the 36 samples positive on the pan-*Chlamydiales* PCR or *W. chondrophila* PCR (see Supplementary material, Table S3). This included 11 of the 14 *C. trachomatis* PCR-positive samples, two of the five *W. chondrophila* PCR positives (both also positive on the pan-*Chlamydiales* PCR), and 12 of the 17 samples positive for other *Chlamydiales*. All sequences were of sufficient quality to allow them to be classified according to the genus level.

Of the 14 samples that were pan-*Chlamydiales* PCR positive and *C. trachomatis* PCR negative, 11 samples (79%) had their best sequence match with sequences detected in respiratory tract samples. Seven of these samples contained sequences that have been associated with chest infections in children. They comprised five samples with sequences that were 100% identical to a *Chlamydia* spp. (uncultured *Chlamydiales* bacterium clone VS30013), one with a sequence identical to a *Parachlamydia* spp. (VS30055), both previously detected in the nasopharynx of children with pneumonia [12]; and one sample with 96% match with the common respiratory pathogen *Chlamydia pneumoniae* (and 94% match with *C. abortus*). The remaining four samples comprised three with a best match to *Neochlamydia* spp. and one to *Parachlamydia* spp. [12], [16], [17].

The two sequences obtained for the *W. chondrophila* PCR-positive samples matched 100% the *Chlamydia* spp. sequence VS30013. Direct sequencing of the *W. chondrophila* amplicons failed in all five positive samples, possibly because of the short amplicon.

## Discussion

### Prinicipal findings

Four percent of women had urine samples positive for a range of *Chlamydiale*s, including species known to be associated with respiratory infections in children [12], but we did not find any significant associations with adverse pregnancy outcome.

### Strengths and limitations

This is the first study applying the pan-*Chlamydiales* assay to genitourinary samples from pregnant women. It is also the largest study of *Chlamydiales* in early pregnancy to date and the only community-based study. We have detailed information on the characteristics of the women, and extremely high rates of follow up at 16 weeks and term. The women provided samples very early in pregnancy (mean 49 days gestation), and came from a wide range of ages, social classes and ethnic groups.

The sample size was small for associations but not for prevalence. Hence, the main limitation was the lack of power to explore possible associations between infection and adverse pregnancy outcome due to the relatively small numbers of women with *Chlamydiales*, miscarriage or preterm birth. However, this was an exploratory study of an existing cohort and was limited by the number of stored samples available. As these PCRs have never been used in a community-based population we did not know what prevalence of *Chlamydiales* to expect or how much they might increase the risk of adverse pregnancy outcomes.

Vaginal samples might have had higher rates of more relevant infections than urine samples. However, vaginal samples were not stored after analysis for bacterial vaginosis [6], and other studies suggest that urine sampling for *C. trachomatis* in pregnancy may be equivalent to endocervical sampling [9]. Although it is likely that some DNA degradation had occurred due to prolonged storage, all the samples that had tested positive for *M. genitalium* in 2002 remained positive when retested in 2011. Urine samples were unavailable for 30% of the cohort, partly because of loss of identifiers linking to the outcome data. However, characteristics of women included in the study were similar to those not included.

Another weakness is the failure to confirm the five *W. chondrophila* PCR-positive samples, either by direct sequencing from the PCR amplicon (0/5 confirmed) or from the pan-*Chlamydiales* PCR (3/5 confirmed). It is unclear whether this reflects lack of specificity of the *W. chondrophila* PCR, or the presence of multiple *Chlamydiales* species in the pan-*Chlamydiales*-positive samples, which might have been preferentially amplified and consequently detected by sequencing.

Even with the use of internal sequencing primers, (which tend to improve the quality of the sequencing significantly as the influence of PCR-generated artefacts is minimized), the quality of the sequences was not always sufficient to yield full-length sequences between the primers. Only unambiguous sequences were used for the database search and the percentages of homology of the sequence and the nearest match is given in the Supplementary material (Table S3). Short 16S rRNA gene sequences such as the approximately. 160 bp in the present study often limit the species identification due to sequence similarity between species. However, in the present study several sequences had no perfect match in the database. These sequences may represent new species or PCR-generated sequence variation as a result of the low amount of organisms present in the sample. Lastly, in contrast to vaginal or placental samples, *W. chondrophila* has almost never been identified in urine samples, with only one case identified in two studies including almost 800 women [4], [18].

Although the mean gestation of 49 days at recruitment means very early miscarriages might be missed, such miscarriages are not generally thought to be associated with infection [1]. Finally, apart from bacterial vaginosis [10] and *Mycoplasma genitalium* [7], we did not investigate other possible infectious causes of preterm birth. We also lacked details on antibiotic use.

### Comparison with other studies

Two serological case–control studies from the UK and Switzerland suggested past infection with *W. chondrophila* was associated with miscarriage [3], [4]. In the study from London, anti-*W. chondrophila* IgG titres >1:64 were found in 32% of 69 women with miscarriage and 7% of 169 pregnant control women (p <0.001). Although evidence of *W. chondrophila* infection has been found in the placenta of women with miscarriage [4], [19], no studies have shown a significant association between miscarriage and current infection as shown by positive PCR. Hence, it may not be surprising that the present study based on PCR analysis of urine samples does not show an association with adverse pregnancy outcomes.

Other *Chlamydiales* such as *Parachlamydia acanthamoebae* might also contribute to adverse pregnancy outcome [20]. In a related study, none of 169 women with uneventful pregnancies had positive serology for *Parachlamydia* compared with 2.6% (7) of 269 women with miscarriage (p <0.05) [5]. In our study three samples were positive for *Parachlamydia* species on sequencing. By contrast, Romero *et al.* found no difference in the vaginal microbiota of pregnant women who subsequently had a preterm birth and those who delivered at term [21]. However, they did not look specifically for *Chlamydiales*.

Although *C. trachomatis* infection may be associated with miscarriage and/or preterm birth [22], [23], results are conflicting [1], [24]. Two large studies found an association between urogenital *C. trachomatis* and preterm birth [18], [23]. In both studies the prevalence of *C. trachomatis* (3%–4%) was higher than in our sample. Current or past infection with *Chlamydiales* could affect placental integrity and cause adverse pregnancy outcomes through inflammatory or immune mechanisms [1]. In our study, two of three women positive on both the *Chlamydiales and W. chondrophila* PCRs had an adverse pregnancy outcome. Higher load of bacteria might be associated with a greater inflammatory or immune response. Finally, unlike others [25], we did not find *C. trachomatis* infection was higher among smokers, but the number of women who reported smoking during pregnancy (*n* = 48) was small.

### Implications

This community-based study found that one in 25 relatively low-risk, multi-ethnic, newly pregnant women (mean age 31 years) had urogenital *Chlamydiales*. Although it did not clarify whether *Chlamydiales* are an important cause of adverse pregnancy outcome, the findings are novel and would be very useful to anyone planning a definitive cohort study or trial of screening. It is possible that *W. chondrophila* might be associated with preterm birth, but the detection rate (<1%) was very low in this urban, community-based population. Increased rates might be found in vaginal samples from higher risk women. Future studies might explore whether women with a history of recurrent miscarriage or preterm birth should be tested for urogenital C*hlamydiales.*

It is unclear how some of these infections are acquired. Zoonotic infections like *C. abortus* occur occasionally in farm workers, and women may be advised to avoid contact with ruminants during pregnancy [5]. *Chlamydia trachomatis* is a common sexually transmitted infection and annual testing is recommended for sexually experienced women aged <25 years. Three of the sequences from the *Chlamydiales*-positive samples matched sequences obtained from environmental sources. Whether these reflect contamination from tap-water, gardening or other external sources [17] or actual infection of the patient remains unclear. Current advice is that pregnant women should wash their hands after contact with soil or animals and before each meal [26].

Finally, the relatively high proportion of urogenital *Chlamydiales* associated with respiratory infections in neonates and children (28%, seven of 25 samples sequenced) is interesting. It suggests that vertical transmission may be possible: babies of infected women might become infected with *Chlamydiales* as they pass through the birth canal during childbirth, potentially leading to pneumonia. It is also possible that pregnant women might become colonized with *Chlamydiales* associated with paediatric respiratory infections from unknown environmental sources. This might include contact with young children.

## Funding

Funding was received from the UK Medical Research Council, grant number MR/K027050/1.

## Transparency declaration

All authors declare that they have no conflicts of interest.

## Figures and Tables

**Table 1:** *Chlamydiales* related to demographics of 847 newly pregnant women

Characteristic	Number with characteristic	Any *Chlamydiales* (*n* = 36)	*Waddlia chondrophila* (*n* = 5)	*Chlamydia trachomatis* (*n* = 14)	*Other Chlamydiales* (*n* = 17) excluding *W. chondrophila and C. trachomatis*
Age (years)					
<20	30	4	0	4^a^	0
20–24	76	5	0	4^a^	1
25–37	683	26	5	6^a^	15
38+	58	1	0	0^a^	1
Black	78	8	0	6^a^	2
Not Black	689	20	4	5^a^	11
Single	60	4	0	3	1
Has partner	708	24	4	8	12
Social class 1–2^b^	442	12	3	3	6
Social class 3–5^b^	292	14	1	6	7
Condoms	229	9	2	3	4
Other/no contraception	533	17	2	6	9
Smoked in pregnancy	48	0	0	0	0
Did not smoke	433	15	3	4	8
History of miscarriage	161	4	0	2	2
No history of miscarriage	605	24	4	9	11
History of preterm birth	28	0	0	0	0
No history of preterm birth	737	27	4	10	13
Bacterial vaginosis	123	9	0	7^a^	3
No bacterial vaginosis	674	25	5	6^a^	13

a Infection with *C. trachomatis* was more common in women aged< 25, of black ethnicity or with bacterial vaginosis (p <0.001).

b Social class based on occupation [6], [15]. For women who were unemployed or students, partner's social class was used. 1, professional; 2, managerial and technical; 3, skilled manual or non-manual; 4, partly skilled; 5, unskilled.

**Table 2:** Detailed characteristics of the five women with *Waddlia chondrophila*-positive samples

Age in years	27	31	32	29	33
Ethnicity	White	White	Asian	White	Unknown
Social class^a^	3	1	2	2	Unknown
Partner status	Married/ cohabiting	Married/ cohabiting	Married/ cohabiting	Married/ cohabiting	Unknown
Condoms	No	Yes	No	Yes	Unknown
Smoked in pregnancy	Unknown	No	No	No	Unknown
History of miscarriage	No	No	No	No	Unknown
History of preterm birth	No	No	No	No	Unknown
*Chlamydia trachomatis*	Neg	Neg	Neg	Neg	Neg
Bacterial vaginosis	Neg	Neg	Neg	Neg	Neg
*Mycoplasma genitalium*	Neg	Neg	Neg	Neg	Neg

a Social class based on occupation [6], [15]. For women who were unemployed or students, partner's social class was used. 1, professional; 2, managerial and technical; 3, skilled manual or non-manual; 4, partly skilled; 5, unskilled.

**Table 3:** Rate of miscarriage at ≤12 weeks gestation in 827 infected and uninfected women

	Miscarriage rate in infected women	Miscarriage rate in uninfected women	p
Any *Chlamydiales* *n* = 35	3/35 (9%)	78/792 (10%)	1.00
*Waddlia chondrophila* *n* = 4^a^	1/4 (25%)	80/823 (10%)	0.34
*Chlamydia trachomatis* *n* = 14	0/14 (0%)	81/813 (10%)	0.38
*Other Chlamydiales* (not *W. chondrophila* or *C. trachomatis*) *n* = 17	2/17 (12%)	79/810 (10%)	0.68

a The fifth *W. chondrophila*-positive woman had a termination of pregnancy at 11 weeks.

**Table 4:** Rate of spontaneous preterm birth at <37 weeks gestation in 628 infected and uninfected women

	Preterm birth rate in infected women	Preterm birth rate in uninfected women	p
Any *Chlamydiales* *n* = 27	2/27 (7%)	21/601 (3%)	0.26
*Waddlia chondrophila* *n* = 3	1/3 (33%)	22/625 (4%)	0.11
*Chlamydia trachomatis* *n* = 11	1/11 (9%)	22/617 (4%)	0.34
*Other Chlamydiales* (not *W. chondrophila* or *C. trachomatis*) *n* = 13	0/13 (0%)	23/615 (4%)	1.00

## Appendix A Supplementary data

The following supplementary material is available for this article:

**Table S1**. Characteristics of 847 included and 369 excluded women (with no linked samples or insufficient samples for analysis)

**Table S2**. Characteristics of five women infected with chlamydia related bacteria who had a miscarriage (*n* = 3) or spontaneous preterm birth (*n* = 2)

**Table S3**. Sequencing of 36 samples positive on the Pan-*Chlamydiales* or *Waddlia chondrophila* PCR

## References

[1] Baud D., Regan L., Greub G. (2008). Emerging role of *Chlamydia* and *Chlamydia*-like organisms in adverse pregnancy outcomes. Curr Opin Infect Dis.

[2] Wheelhouse N., Mearns R., Willoughby K., Wright E., Turnbull D., Longbottom D. (2015). Evidence of members of the *Chlamydiales* in bovine abortions in England and Wales. Vet Rec.

[3] Baud D., Thomas V., Arafa A., Regan L., Greub G. (2007). *Waddlia chondrophila*, a potential agent of human fetal death. Emerg Infect Dis.

[4] Baud D., Goy G., Osterheld M.C., Croxatto A., Borel N., Vial Y. (2014). Role of *Waddlia chondrophila* placental infection in miscarriage. Emerg Infect Dis.

[5] Baud D., Greub G. (2011). Intracellular bacteria and adverse pregnancy outcomes. Clin Microbiol Infect.

[6] Oakeshott P., Hay P., Hay S., Steinke F., Rink E., Kerry S. (2002). Association between bacterial vaginosis or chlamydial infection and miscarriage before 16 weeks' gestation: prospective community based cohort study. BMJ.

[7] Oakeshott P., Hay P., Taylor-Robinson D., Hay S., Dohn B., Kerry S. (2004). Prevalence of *Mycoplasma genitalium* in early pregnancy and relationship between its presence and pregnancy outcome. BJOG.

[8] Oakeshott P., Hay P., Hay S., Steinke F., Thomas B., Rink E. (2002). Detection of *Chlamydia trachomatis* infection in early pregnancy using self-administered vaginal swabs and first pass urines: a cross sectional community based survey. Br J Gen Pract.

[9] Roberts S.W., Sheffield J.S., McIntire D.D., Alexander J.M. (2011). Urine screening for *Chlamydia trachomatis* during pregnancy. Obstet Gynecol.

[10] Oakeshott P., Kerry S., Hay S., Hay P. (2003). Bacterial vaginosis and preterm birth: prospective community based cohort study. Br J Gen Pract.

[11] Goy G., Croxatto A., Posfay-Barbe K.M., Gervaix A., Greub G. (2009). Development of a real-time PCR for the specific detection of *Waddlia chondrophila* in clinical samples. Eur J Clin Microbiol Infect Dis.

[12] Lienard J., Croxatto A., Aeby S., Jaton K., Posfay-Barbe K., Gervaix A. (2011). Development of a new chlamydiales-specific real-time PCR and its application to respiratory clinical samples. J Clin Microbiol.

[13] Jensen J.S., Bjornelius E., Dohn B., Lidbrink P. (2004). Use of TaqMan 5 nuclease real-time PCR for quantitative detection of *Mycoplasma genitalium* DNA in males with and without urethritis who were attendees at a sexually transmitted disease clinic. J Clin Microbiol.

[14] Westh H., Jensen J.S. (2008). Low prevalence of the new variant of *Chlamydia trachomatis* in Denmark. Sex Transm Infect.

[15] Office for National Statistics. 2000 (2000). Standard occupational classification 2000 (SOC2000).

[16] Haider S., Collingro A., Walochnik J., Wagner M., Horn M. (2008). *Chlamydia*-like bacteria in respiratory samples of community-acquired pneumonia patients. FEMS Microbiol Lett.

[17] Corsaro D., Venditti D. (2015). Detection of novel Chlamydiae and Legionellales from human nasal samples of healthy volunteers. Folia Microbiol (Praha).

[18] Baud D., Goy G., Vasilevsky S., Osterheld M.C., Roth-Kleiner M., Croxatto A. (2015). Roles of bovine *Waddlia chondrophila* and *Chlamydia trachomatis* in human preterm birth. New Microbes New Infect.

[19] Baud D., Goy G., Osterheld M.C., Borel N., Vial Y., Pospischil A. (2011). *Waddlia chondrophila*: from bovine abortion to human miscarriage. Clin Infect Dis.

[20] Baud D., Goy G., Gerber S., Vial Y., Hohlfeld P., Greub G. (2009). Evidence of maternal-fetal transmission of *Parachlamydia acanthamoebae*. Emerg Infect Dis.

[21] Romero R., Hassan S.S., Gajer P., Tarca A.L., Fadrosh D.W., Bieda J. (2014). The vaginal microbiota of pregnant women who subsequently have spontaneous preterm labor and delivery and those with a normal delivery at term. Microbiome.

[22] Baud D., Goy G., Jaton K., Osterheld M.C., Blumer S., Borel N. (2011). Role of *Chlamydia trachomatis* in miscarriage. Emerg Infect Dis.

[23] Rours G.I., Duijts L., Moll H.A., Arends L.R., de G.R., Jaddoe V.W. (2011). *Chlamydia trachomatis* infection during pregnancy associated with preterm delivery: a population-based prospective cohort study. Eur J Epidemiol.

[24] Howie S.E., Horner P.J., Horne A.W. (2011). *Chlamydia trachomatis* infection during pregnancy: known unknowns. Discov Med.

[25] Hwang L.Y., Ma Y., Moscicki A.B. (2014). Biological and behavioral risks for incident *Chlamydia trachomatis* infection in a prospective cohort. Obstet Gynecol.

[26] NICE (2016). Antenatal care for uncomplicated pregnancies. NICE guidelines CG62.

