# Supplementary material for: Chlamydia related bacteria (Chlamydiales) in early pregnancy: community-based cohort study
Source: Clin Microbiol Infect. 2017 Feb;23(2):119.e9–119.e14. doi: 10.1016/j.cmi.2016.10.011 (PMC5317141; doi:10.1016/j.cmi.2016.10.011)
Supplement: Supplementary file 1 [file mmc1.doc]

**Table S1 Characteristics of 847 included and 369 excluded women (with no linked samples or insufficient samples for analysis)**

|  | **Total number** | **Included**  **N=847** | | **Excluded**  **N=369** | |
| --- | --- | --- | --- | --- | --- |
|  |  |  |  |  |  |
| Age <20 | 42 | 30 | 3.5% | 12 | 3.3% |
| 20-24 | 110 | 76 | 9.0 | 34 | 9.2% |
| 25-37 | 971 | 683 | 80.6% | 288 | 78.0% |
| 38+ years | 93 | 58 | 6.8% | 35 | 9.5% |
|  |  |  |  |  |  |
| Black ethnicity | 119 | 78 | 10.2% | 41 | 12.1% |
| Other ethnic group | 988 | 689 | 89.8% | 299 | 87.9% |
|  |  |  |  |  |  |
| Single | 96 | 60 | 7.8% | 36 | 10.7% |
| Has partner | 1009 | 708 | 92.2% | 301 | 89.3% |
|  |  |  |  |  |  |
| Social class 1-2* | 627 | 442 | 60.2% | 185 | 59.2% |
| Social class 3-5* | 419 | 292 | 39.8% | 127 | 40.8% |
|  |  |  |  |  |  |
| Condoms | 313 | 229 | 30.1% | 84 | 25.1% |
| Other/no contraception | 783 | 533 | 69.9% | 250 | 74.9% |
|  |  |  |  |  |  |
| Smoked in pregnancy | 75 | 48 | 10.0% | 27 | 14.2% |
| Did not smoke | 596 | 433 | 90.0% | 163 | 85.8% |
|  |  |  |  |  |  |
| History of miscarriage | 228 | 161 | 21.0% | 67 | 19.5% |
| No history of miscarriage | 881 | 605 | 79.0% | 276 | 80.5% |
|  |  |  |  |  |  |
| History of preterm birth | 39 | 28 | 3.7% | 11 | 3.2% |
| No history of preterm birth | 1065 | 737 | 96.3% | 328 | 96.8% |
|  |  |  |  |  |  |
| Bacterial vaginosis | 174 | 123 | 15.4% | 51 | 14.7 % |
| No bacterial vaginosis | 973 | 674 | 84.6% | 299 | 85.3% |

*Social class based on occupation [6;15]. For women who were unemployed or students, partner’s social class was used. 1 professional, 2 managerial and technical, 3 skilled manual or non-manual, 4 partly skilled, 5 unskilled

**Table S**2: Characteristics of five women infected with chlamydia related bacteria who had a miscarriage (n=3) or spontaneous preterm birth (n=2)

| Age in years | Ethnicity | Social  Class * | Gestation in weeks at miscarriage or preterm birth | PCR result positive for: | Bacterial  vaginosis  at recruit-  ment^6^ (Nugent’s criteria) | Sequence result |
| --- | --- | --- | --- | --- | --- | --- |
| 27 | White | 3 | 10 | Pan-*Chlamydiales* and *W. chondrophila* | Neg | 100% Uncultured *Chlamydiales* bacterium clone VS30013 |
| 26 | Unknown | Unknown | 12 | Pan-*Chlamydiales* | Neg | 93% Uncultured *Chlamydiales* bacterium clone HE210032biof |
| 31 | Unknown | Unknown | 11 | Pan-*Chlamydiales* | Pos | Sequence failed |
| 32 | Asian | 2 | 36 | Pan-*Chlamydiales* and *W. chondrophila* | Neg | Sequence failed |
| 32 | White | 2 | 34 | Pan-*Chlamydiales* and *C. trachomatis* | Pos | Sequence failed |

*Social class based on occupation [6;15]. For women who were unemployed or students, partner’s social class was used.

1 professional, 2 managerial and technical, 3 skilled manual or non-manual, 4 partly skilled, 5 unskilled.

**Table S3: Sequencing of 36 samples positive on the Pan-*Chlamydiales* or *W.chondrophila* PCR.** Similarity to sequences present in NCBI GenBank is given as percentages of homology of the sequence between the internal sequencing primers and the nearest match. Not all sequences were readable in the full length.

| ***W.chond.* PCR** |  | **Pan-*Chlam.* PCR** | ***C.trach.* PCR** | **Sequencing and taxonomic assignment** |
| --- | --- | --- | --- | --- |
| 0 |  | 1 | 1 | *C. trachomatis* |
| 0 |  | 1 | 1 | *C. trachomatis* |
| 0 |  | 1 | 1 | *C. trachomatis* |
| 0 |  | 1 | 1 | *C. trachomatis* |
| 0 |  | 1 | 1 | *C. trachomatis* |
| 0 |  | 1 | 1 | *C. trachomatis* |
| 0 |  | 1 | 1 | *C. trachomatis* |
| 0 |  | 1 | 1 | *C. trachomatis* |
| 0 |  | 1 | 1 | *C. trachomatis* |
| 0 |  | 1 | 1 | *C. trachomatis* |
| 0 |  | 1 | 1 | *C. trachomatis* |
| 0 |  | 1 | 1 | Sequence failed |
| 0 |  | 1 | 1 | Sequence failed |
| 0 |  | 1 | 1 | Sequence failed |
| 1 |  | 1 | 0 | 100% Uncultured *Chlamydiales* bacterium clone VS30013  *Chlamydia* spp.* ^12^ |
| 1 |  | 1 | 0 | 100% Uncultured *Chlamydiales* bacterium clone VS30013  *Chlamydia* spp.* ^12^ |
| 1 |  | 1 | 0 | Sequence failed |
| 0 |  | 1 | 0 | 100% Uncultured *Chlamydiales* bacterium clone VS30013  *Chlamydia* spp.* ^12^ |
| 0 |  | 1 | 0 | 100% Uncultured *Chlamydiales* bacterium clone VS30013  *Chlamydia* spp.* ^12^ |
| 0 |  | 1 | 0 | 100% Uncultured *Chlamydiales* bacterium clone VS30013  *Chlamydia* spp.* ^12^ |
| 0 |  | 1 | 0 | 96% *Chlamydia pneumoniae*.* |
| 0 |  | 1 | 0 | 94% Uncultured Candidatus *Rhabdochlamydia* sp. clone CN808  *Neochlamydia* spp.*^16^ |
| 0 |  | 1 | 0 | 100% Chlamydiales bacterium NS11  *Neochlamydia* spp.* ^17^ |
| 0 |  | 1 | 0 | 99% Uncultured *Chlamydiales* KK135A0008 (environmental)  *Neochlamydia* spp. |
| 0 |  | 1 | 0 | 94% Uncultured *Chlamydiales* bacterium clone 21IR (environmental)  *Neochlamydia* spp. |
| 0 |  | 1 | 0 | 96% Uncultured *Chlamydiales* bacterium clone GE11053  *Neochlamydia* spp.* ^12^ |
| 0 |  | 1 | 0 | 97% Uncultured *Chlamydiales* bacterium clone HE210023  *Parachlamydia* spp.* ^12^ |
| 0 |  | 1 | 0 | 93% Uncultured *Chlamydiales* bacterium clone HE210032biof (environmental)  *Parachlamydia* spp. |
| 0 |  | 1 | 0 | 100% Uncultured bacterium partial 16S rRNA gene, Mineral.bttm.1.4.1.2_1  98% Uncultured *Chlamydiales* bacterium clone VS30055  *Parachlamydia* spp.* ^12^ |
| 0 |  | 1 | 0 | Sequence failed |
| 0 |  | 1 | 0 | Sequence failed |
| 0 |  | 1 | 0 | Sequence failed |
| 0 |  | 1 | 0 | Sequence failed |
| 0 |  | 1 | 0 | Sequence failed |
| 1 |  | 0 | 0 | Sequence failed |
| 1 |  | 0 | 0 | Sequence failed |

* Sequence with best match in GenBank or with >97% identity found in respiratory tract specimens. 1=positive PCR. 0-negative PCR
